# The Effect of Tumor Characteristics and Location on the Extent of Lymph Node Metastases of Head and Neck Cutaneous Squamous Cell Carcinoma

**DOI:** 10.3389/fonc.2022.874295

**Published:** 2022-05-30

**Authors:** Bram van Leer, Alet J. G. Leus, Boukje A. C. van Dijk, Marloes S. van Kester, Gyorgy B. Halmos, Gilles F.H. Diercks, Bert van der Vegt, Jeroen Vister, Emoke Rácz, Boudewijn E. C. Plaat

**Affiliations:** ^1^ Department of Otorhinolaryngology, Head and Neck Surgery, University Medical Center Groningen, University of Groningen, Groningen, Netherlands; ^2^ Department of Dermatology, University Medical Center Groningen, University of Groningen, Groningen, Netherlands; ^3^ Department of Epidemiology, University Medical Center Groningen, University of Groningen, Groningen, Netherlands; ^4^ Department of Research and Development, Netherlands Comprehensive Cancer Organization Intergraal Kankercentrum Nederland (IKNL), Utrecht, Netherlands; ^5^ Department of Pathology, University Medical Center Groningen, University of Groningen, Groningen, Netherlands; ^6^ Department of Radiology, University Medical Center Groningen, University of Groningen, Groningen, Netherlands

**Keywords:** cutaneous squamous cell carcinoma (cSCC), lymph node metastasis (LNM), metastasis pattern, neck dissection (ND), parotidectomy, primary tumor location (PTL), tumor characteristics, clinical recommendations

## Abstract

**Background:**

The extent of a neck dissection for patients with metastasis of cutaneous squamous cell carcinoma of the head and neck (HNcSCC) is still subject to debate and clear guidelines are lacking. Tumor characteristics like size, differentiation and tumor location are known risk factors for lymph node metastasis (LNM). There is some evidence that, depending on tumor location, LNM follows a specific pattern. This study aims to identify which tumor characteristics can predict the pattern and extent of LNM.

**Method:**

In this cohort study 80 patients were included, who underwent a primary neck dissection for LNM of HNcSCC between 2003 and 2018 at the University Medical Center Groningen, the Netherlands. Retrospective data was collected for primary tumor characteristics and LNM and included surgical and follow-up data. Influence of tumor characteristics on the extent of LNM was analyzed using non-parametric tests. Logistic regression analysis were used to identify a metastasis pattern based on the primary tumor location.

**Results:**

Only primary tumor location was associated with the pattern of LNM. HNcSCC of the ear metastasized to level II (OR = 2.6) and the parotid gland (OR = 3.6). Cutaneous lip carcinoma metastasized to ipsilateral and contralateral level I (OR = 5.3). Posterior scalp tumors showed a metastasis pattern to level II (OR = 5.6); level III (OR = 11.2), level IV (OR = 4.7) and the parotid gland (OR = 10.8). Ear canal tumors showed a low risk of LNM for all levels. The extent of LNM was not related to age or any tumor characteristics i.e. tumor diameter, infiltration depth, differentiation grade, perineural growth and vascular invasion.

**Conclusion:**

Primary tumor location determines the LNM pattern. Whereas known unfavorable tumor characteristics did not relate to the extent of LNM. Location guided limited neck dissection combined with parotidectomy will treat most patients adequately.

## Introduction

The global incidence of cutaneous squamous cell carcinoma (cSCC) in 2017 was estimated at 1.8 million and is expected to increase due to the aging population (1–4). The main localization of cSCC is in areas of skin most exposed to the sun, namely the head and neck region (5, 6). Although the 5-year survival rate of patients with head and neck cSCC (HNcSCC) is over 90%, 2-5% of this growing group of mainly older patients develops lymph node metastasis (LNM). Five-year disease-specific survival rates of <40% for patients with LNM have been reported (1, 7–9).

Risk factors for developing LNM include tumor size, invasion depth, differentiation grade, the presence of perineural growth, bone or vascular invasion, and tumors localized at specific regions of the face (i.e. ear or lip) (4, 8–12).

In cases of regional LNM, a neck dissection of ipsilateral cervical lymph nodes is advised, depending on the primary site including removal of the parotid gland (13, 14). The extent of the advised neck dissection (i.e. selective neck dissection) with or without parotidectomy is subject to debate and no clear guidelines have been provided. Some association has been shown between the localization of the cSCC and the locoregional metastases. However, sufficient evidence for a selective neck dissection was only provided for HNcSCC localized at the anterior face (15, 16).

Limiting the extent of a neck dissection could decrease morbidity, duration of surgery and reduce the risk of complications, especially in old or frail patients (17, 18). Advice regarding the extent of a neck dissection based on tumor characteristics could therefore be helpful. The aim of the study was to assess the effect of the primary tumor location and characteristics on the location and extent of the regional metastasis in order to give a recommendation for a more selective neck dissection.

## Methods

For this retrospective study, all consecutive patients who underwent a primary neck dissection with or without a parotidectomy for LNM of HNcSCC between 2003 and 2018 in the University Medical Center Groningen (UMCG), the Netherlands, were included. Patients were considered to have LNM by a multi-disciplinary group of head and neck specialists based on imaging, clinical findings and biopsy. For patients with a history of multiple primary HNcSCC, the index tumor was identified. Since >95% of the cSCC metastasize within the first two years, the index tumor was determined based on the time between onset of the primary tumor and LNM as well as tumor location and risk factors (8, 9). Patients were excluded in cases of earlier ipsilateral neck dissection, non-cutaneous head and neck squamous cell carcinoma in the past 20 years, with no information available for the primary tumor, or when an index tumor could not be identified.

The Institutional Review Board of the University Medical Center Groningen assessed this retrospective study (IRB research register nr 202000804) and judged that there was no need for approval based on the Dutch Medical Research Law (Wet medisch-wetenschappelijk onderzoek met mensen [WMO]).

Patient and tumor characteristics were collected from electronic patient files and histopathology reports (**Table 1**). In cases of missing data, clinical reports were used. Tumor localization was classified according to Vauterin et al. (15) Tumor location was determined from clinical reports and, when not clearly specified, from clinical photographs. The authors (re)staged the primary tumor according to three different classification systems: the Brigham and Women’s Hospital (BWH), the American Joint Committee on Cancer 7^th^ edition (AJCC 7) and the American Joint Committee on Cancer 8^th^ edition (AJCC 8) classifications (4, 19).

**Table 1:** Patient and index tumor characteristics.

Gender	n (%)	AJCC 7	n (%)
Men	65 (81.3)	T1	12 (15.0)
Women	15 (18.8)	T2	60 (75.0)
		T3	3 (3.8)
Age	years (range)	T4	2 (2.5)
Median	75.5 (40 - 91)	Unknown	3 (3.8)
History of HNcSCC	n (%)	AJCC 8	n (%)
Yes	28 (35.0)	T1	25 (31.3)
No	52 (65.0)	T2	14 (17.5)
		T3	34 (42.5)
Treatment of primary tumor	n (%)	T4a	2 (2.5)
Surgery	39 (48.8)	T4b	1 (1.3)
Surgery + Radiotherapy	31 (38.8)	Unknown	4 (5.0)
Radiotherapy	10 (12.5)		
		BWH	n (%)
Excision margins	n (%)	T1	18 (22.5)
Clear margin	43 (53.8)	T2a	25 (31.3)
Positive margin	26 (32.5)	T2b	29 (36.3)
Unknown	1 (1.3)	T3	5 (6.3)
		Unknown	3 (3.8)
Tumor diameter	mm (range)		
Mean	25.9 (4.0 - 70.0)	Tumor location	n (%)
Unknown	n = 7	Ear	18 (22.5)
		Cutaneous lip	12 (15.0)
Tumor infiltration	mm (range)	Superior	2 (2.5)
Mean	7.1 (1.1 - 40.0)	Inferior	8 (10)
Unknown	n = 2	Commissure	2 (2.5)
		Temporal scalp	11 (13.8)
Tumor differentiation	n (%)	Posterior scalp	9 (11.3)
Well	16 (20.0)	Ear canal	7 (8.8)
Moderate	46 (57.5)	Postauricular	6 (7.5)
Poor	18 (22.5)	Nose	5 (6.3)
		Frontal scalp	4 (5.0)
Perineural invasion	n (%)	Cheeks	4 (5.0)
Yes	22 (27.5)	Anterior scalp	2 (2.5)
No	58 (72.5)	Neck	1 (1.3)
		Chin	1 (1.3)
Vascular invasion	n (%)	Periorbital	0 (0.0)
Yes	7 (8.8)		
No	73 (91.3)		

HNcSCC, Head and Neck cutaneous squamous cell carcinoma; AJCC 7, the American Joint Committee on Cancer 7^th^ edition; AJCC 8, the American Joint Committee on Cancer 8^th^ edition; BWH, Brigham and Women’s Hospital.

Location of the LNM was categorized according to the anatomical distribution: level I-V, the parotid gland and the postauricular or occipital region. The histopathological results for each nodal level were obtained from pathological reports, which were reported per location. To assure a distinction between level II and parotid intraglandular lymph nodes, in 11 patients histopathological slides were re-assessed by an experienced head and neck pathologist to verify the results. Recurrence-free survival was defined as the time between date of surgery and date of recurrent disease, death or last checkup. Survival was defined as years alive after primary tumor diagnosis. For patients without a (modified) radical neck dissection, untreated neck levels were considered positive or negative depending on whether the patient had a 2-year recurrence-free period. When patients were lost to follow-up within 2 years these levels were scored as unknown. Metastases with clear cutaneous involvement were categorized as in-transit metastasis and not included as LNM. Contralateral lymph nodes were scored separately and only included in analysis using the number of involved levels.

For statistical analysis SPSS 23 (IBM SPSS Statistics for Windows, Version 23.0. Armonk, USA) was used. Figures were created using Excel 2010 (Microsoft Corporation; Redmond, USA) and SPSS 23. The effect of tumor characteristics (i.e. known risk factors) on the number of histopathologically positive lymph nodal levels was analyzed using an ordinal regression analysis for all factors except age. In this case the test of parallel lines showed to be significant and a multinominal regression analysis was used instead. To deal with small numbers in groups, five or more involved lymph nodal levels were combined as one. Disease-specific survival was analyzed using Kaplan-Meier analysis for respectively ≤ 2 and > 2 lymph nodal levels involved based upon histopathology only (i.e. without 2 year follow-up). Log rank test was used for significance. A risk-ratio was defined as the distribution of LNM per tumor location. Due to small numbers, sublevels were not included for primary tumor location and LNM. The odds that an individual lymph node level was involved in the pattern of metastasis was calculated using a logistic regression analysis. As an equal distribution for each location in our study population would have been 6, the odds-ratio was calculated only for tumor locations with more than 5 patients. A p-value of <0.05 was considered statistically significant.

## Results

Of the 93 selected patients, 80 were included in the study. Six patients were excluded because there was no information available of the primary tumor. Five were excluded because the neck dissection was not performed in our institute. For 2 patients, the index tumor could not be determined. Twenty-eight (35%) of the included patients were found to have had a previous HNcSCC (other than the index tumor) in their medical history.

As shown in **Table 1**, mean diameter and infiltration depth were respectively 25.9 and 7.1 mm and most index tumors were moderately differentiated (58%). Perineural or vascular invasion occurred in respectively 22 (28%) and 7 (9%) patients. The most common tumor classification for AJCC 7, 8 and BWH were respectively T2 (75%), T3 (43%) and T2b (36%). The majority of the tumors were located on the ears (23%), cutaneous lip (15%) or in the temporal region (14%).

**Figure 1:**
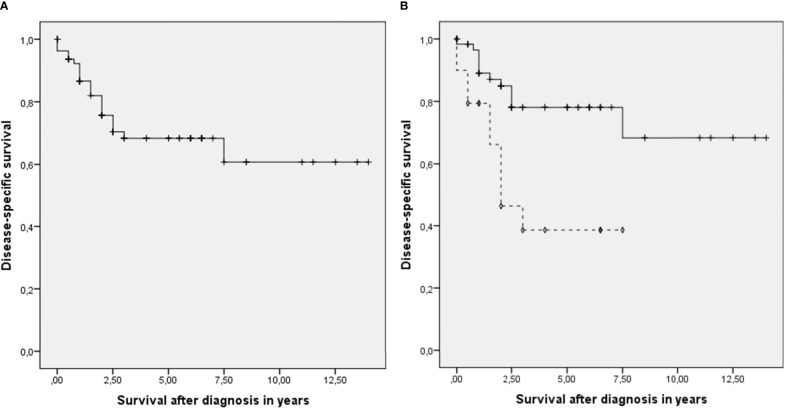
**(A)** Disease-specific survival for all patients with metastasis of head and neck cutaneous squamous cell carcinoma. + censored. **(B)** Disease-specific survival divided between patients ≤ 2 (—) histopathological positive lymph nodal levels at time of surgery and patients with > 2 (- - -). +, ◊ censored.

Ipsilateral (modified) radical neck dissection (i.e. level I to V) combined with a parotidectomy was performed in 32.5% of the cases. LNM were found mostly in the parotid gland (42.5%) and level II (42.5%) (**Table 2**). Contralateral neck dissections were performed in 12 patients, most often when the tumor was localized at the cutaneous lip (42%) or the nose (25%). Twelve patients developed new LNM within 2 years. In 57 patients (71.3%), 2 or less levels were involved (mean 2.1, SD 2.1). At time of surgery this was the case for 60 patients (75%, mean 1.8, SD 1.6). The median overall follow-up period was 2.5 years (range: 0 - 14 years, mean 3.59, SD: 3.3). Mean overall survival was 3.5 years (SD 3.3 years). Mean disease-specific survival for all patients was 9.5 years (CI 95% 7.9 – 11.2 years) (**Figure 1A**). Patients with more than 2 lymph nodal levels involved had a significant higher change of dying (estimated mean > 2 levels: 3.8 years (95% CI 2.3 – 5.3 years) and ≤ 2 levels: 10.6 years (95% CI 8.9 – 12.4 years); p = 0.002) (**Figure 1B**).

**Table 2:** Ipsilateral outcome per lymph nodal level during histopathological examination or during clinical follow-up within 2 years by physical examination or imaging.

	I		II		III		IV		V		Parotid Gland		Post-auricular		Occipital	
Positive																
Neck dissection	19	(23.8)	33	(41.3)	22	(27.5)	12	(15.0)	13	(16.3)	34	(42.5)	4	(5.0)	2	(2.5)
Clinically, during FU	2	(2.5)	1	(1.3)	1	(1.3)	0	(0.0)	1	(1.3)	0	(0.0)	4	(5.0)	1	(1.3)
* Total*	*21*	(26.3)	*34*	(42.5)	*23*	(28.8)	*12*	(15.0)	*14*	(17.5)	*34*	(42.5)	*8*	(10.0)	*3*	(3.8)
Negative																
Neck dissection	36	(45.0)	43	(53.8)	50	(62.5)	34	(42.5)	38	(47.5)	21	(26.3)	0	(0.0)	1	(1.3)
Clinically, during FU	9	(11.3)	1	(1.3)	2	(2.5)	18	(22.5)	15	(18.8)	10	(12.5)	36	(45.0)	38	(47.5)
* Total*	*45*	(56.3)	*44*	(55.0)	*52*	(65.0)	*52*	(65.0)	*53*	(66.3)	*31*	(38.8)	*36*	(45.0)	*39*	(48.8)
Unknown	14	(17.5)	2	(2.5)	5	(6.3)	16	(20.0)	13	(16.3)	15	(18.8)	36	(45.0)	38	(47.5)

Tumors unable to classify due to follow-up period < 2 years or missing pathology were classified as unknown. (% of total number of patients, n = 80).

**Figure 2:**
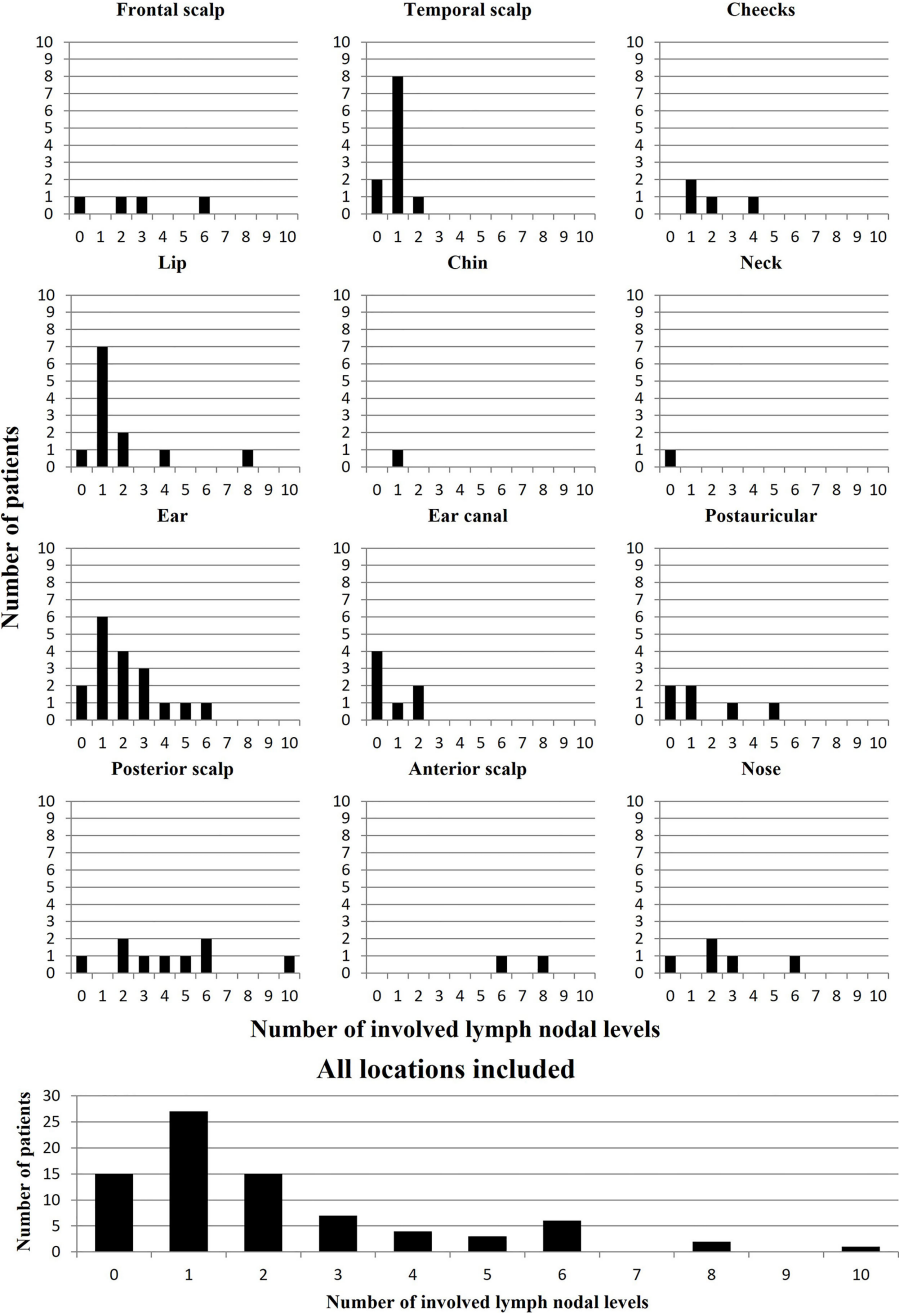
Distribution figure of the total number of patients per total number of involved lymph nodal levels. Depicting the extent of the lymph nodal metastasis per primary tumor location, as well as for all locations together (i.e. tumors at the ear: 1 patient had no metastasis, 7 patients had 1 level involved, 2 had 2 levels involved, etc.) A maximum of 16 metastatic lymph node sites/levels could be involved (i.e. ipsi- and contralateral involvement of neck level I to V, the parotid gland, post-auricular and occipital).

The number of histologically positive lymph nodal levels was not associated (p > 0.05) with stage, age or known risk factors for metastasis, i.e. diameter, infiltration, differentiation, perineural invasion, vascular invasion and excision margins, nor did BWH, AJCC 7 and AJCC 8 stage. To show the extent of the lymph nodal metastasis per primary tumor location, as well as for all locations together (which was used for ordinal regression analysis) **Figure 2** was depicted.

**Figure 3:**
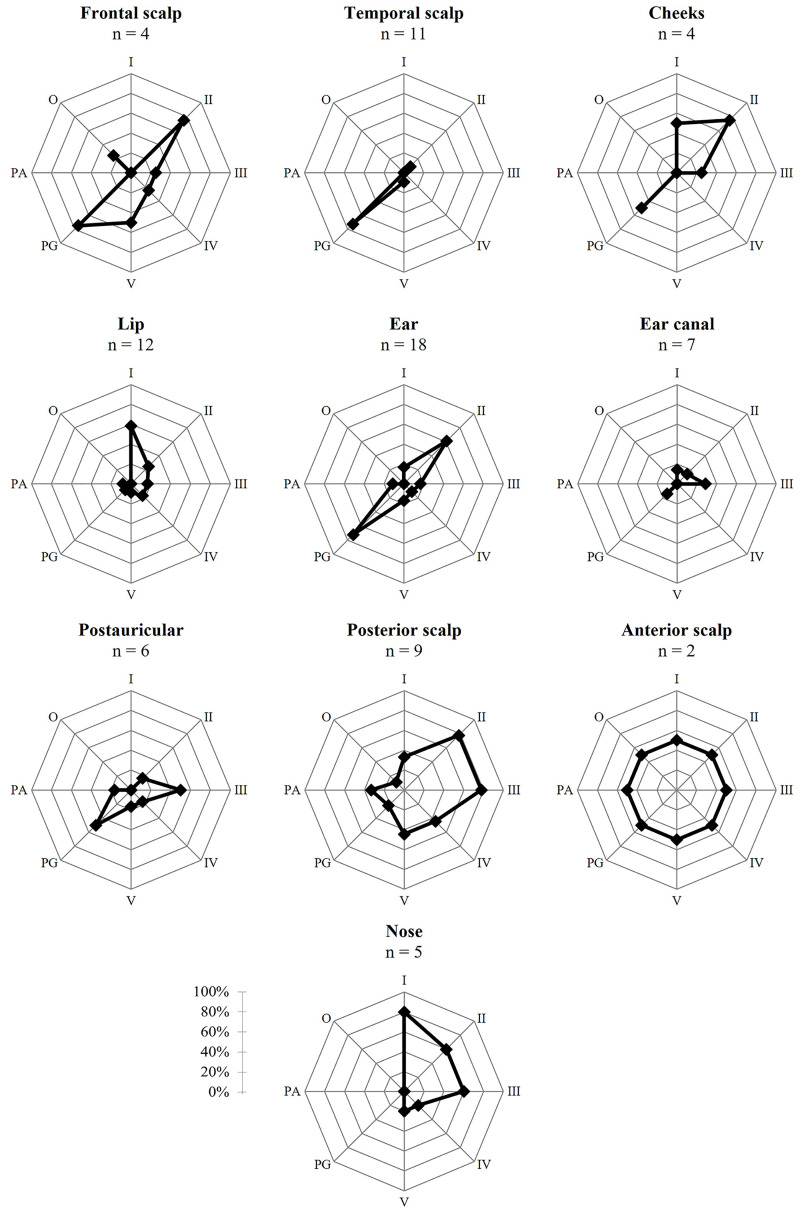
Risk ratio in percentages of tumor metastasis per location per level. Locations with n = 1 are not included; I, level 1; II, level 2; III, level 3; IV, level 4; V, level 5; PG, parotid gland; PA, postauricular; O, occipital.

Tumor localization showed a strong association with the LNM pattern (**Figure 3** and **Table 3**). Tumors localized at the temporal scalp metastasized solely to the parotid gland, whereas localization on the external ear and postauricular skin metastasized to the parotid gland, neck levels II and III. LNM of cutaneous lip carcinoma were mainly localized in level I and II. Separating the superior, inferior and commissure of the lips showed the following distribution: none of the superior lip metastasis involved level IA, in one case level 1B and the other case had no involvement of level I at all. Inferior lip metastasis involved level IA in one case, level IB in 5 cases and no involvement of level I was seen in 2 patients. For one tumor localized at the commissure involvement of level I is unknown at all and for the other patient it was not possible to distinguish retrospectively between level IA and IB. However, due to small numbers we decided not to differentiated any further between subgroups for statistical analysis, as described in the method section.

**Table 3:** Odds ratio of lymph node metastases per location ( > 5 patients) per level ipsilateral.

	Ear	Temporal scalp	Cutaneous lip	Postauricular	Posterior scalp	Ear canal
I	0,42 (0,22)	NA	5,25 (0,02*)	NA	1,75 (0,49)	0,72 (0,78)
II	2,60 **(0,08)**	0,12 **(0,05)**	0,39 (0,18)	0,24 (0,20)	5,57 (0,04*)	0,20 (0,14)
III	0,38 (0,16)	NA	0,41 (0,28)	2,5 (0,29)	11,16 (<0,01*)	1,17 (0,87)
IV	0,60 (0,54)	NA	1,29 (0,77)	0,86 (0,89)	4,7 (0,045*)	NA
V	0,84 (0,81)	0,60 (0,65)	0,43 (0,45)	0,74 (0,79)	3,84 **(0,08)**	NA
PG	3,60 (0,03*)	4,92 **(0,06)**	0,12 **(0,05)**	1 (1,00)	0,47 (0,40)	**0,14 (0,08)**
PA	1,25 (0,81)	NA	0,76 (0,81)	0,94 (0,96)	10,8 (0,02*)	NA
O	NA	NA	NA	NA	6 (0,19)	NA

Likelihood-ratio used for significance. I, level 1; II, level 2; III, level 3; IV, level 4; V, level 5; PG, parotid gland; PA, postauricular; O, occipital; NA, Not applicable; no positive levels found; OR (p value)

*p = < 0.05; in bold p = < 0.1.

No specific LNM pattern was seen for tumors located on the anterior and posterior scalp. Tumors originating from the ear canal showed almost no cervical metastasis. Since the number of tumors localized at the frontal scalp, periorbital skin, chin, cutis of the neck, cheeks and nose was too low, the pattern of LNM originating from these locations could not be assessed. In general, metastases in the occipital lymph nodes were seen in 3 cases (3.8%).

## Discussion

The increased incidence of cSCC in a growing elderly population and its related rise in number of patients presenting with LNM, necessitates a redefinition of the extent of surgical treatment of LNM. Our aim was to investigate the influence of well-known risk factors for LNM in HNcSCC and tumor localization on the extent and pattern of metastatic spread in the neck area. To our knowledge, this is the first study investigating the effect of known risk factors on the extent of regional metastasis. In this retrospective study, these well-known risk factors for LNM, as well as age, did not correlate with the extent of the metastases. Only the location of the tumor proved to be important in relation to the pattern of LNM.

Recent studies showed that a large proportion of HNcSCC patients are considered as frail and these patients are at a higher risk for postoperative complications (17, 20). Since treatment intensity and general anesthesia have shown to be predictors for postoperative complications, reducing the duration and extent of the operation could lower the complication rate and long-term negative effects (17, 18, 21).

None of the tumor characteristics associated with risk for LNM, showed a significant correlation with the number of positive lymph nodal levels. No previous study have explored the effect of the known risk factors on the extent of the LNM (4, 8–11, 22). There is no evidence-based explanation for the absence of correlation; however, one can speculate. The absence of correlation could be explained for several characteristics, by arguing that they influence the possibility of tumor growth into the lymphatic system but do not necessarily predict the growth rate or aggressiveness of the tumor. Surprisingly, no correlation was found with tumor diameter, nor for infiltration or degree of differentiation. One would expect that these characteristics reflecting aggressiveness would influence the extent of LNM. Classification systems, designed to predict prognosis and thereby reflect risk profiles, did not correlate with the extent of LNM either (19, 23). Probably since those are products of the risk factors above. Disease-specific survival analysis showed that there is a correlation between the extent of the metastatic spread and the survival, as has been described earlier in the literature and contributes to the TNM system (12, 24–29). Other studies showed that there is no direct correlation between tumor characteristics and disease specific survival (26, 30, 31). In this study we did not try to predict the chance of lymph node metastasis it self, but we tried to find predictive factors for the extension of metastatic spread. Earlier studies from the same institution which were partly based upon the same database as this study, including cN0 cases, showed similar risk factors for nodal metastasis as studies from other institutions (4, 8–11, 22).

Tumor location could be used to limit the size of the neck dissection. A selective neck dissection of metastases originating from a temporal localized tumor could perhaps be limited to the parotid gland as shown in our study. Furthermore, tumors of the ear were found to be most likely to metastasize to the parotid gland and, to a lesser extent, to level II, and should be included. These findings correspond with the distribution found by Ebrahimi et al. and Vauterin et al. (15, 16).

Interestingly, tumors localized in the ear canal were less likely to metastasize at all. In most cases, after histopathological examination, the parotid gland showed direct invasion of the primary ear canal carcinoma with no signs of LNM. A study of Mi Lee et al. showed that only advanced ear canal cSCC may metastasize to the parotid gland (< 25%), and their metastasis rate is likely to be overestimated (32). This strengthens our advice that, in cases of no clinical signs of other regional metastasis, a parotidectomy alone would be sufficient for adequate treatment.

We showed that tumors of the cutaneous lip are most likely to metastasize to level I and are not likely to metastasize to the parotid gland. Forty two percent metastasized to the contralateral side. Therefore, at least a selective bilateral neck dissection of level I should be performed. To our knowledge, other studies have not included metastasis patterns of specific cutaneous SCC of the lip instead of inclusion of the lip as a whole (15, 16).

A (modified) radical neck dissection without a parotidectomy is advised in the case of localization at the posterior scalp. In our study there was a significantly higher risk for LNM to level II, level III and the postauricular region. Involvement of levels IV and V was also found. Interestingly, Vauterin et al. showed only involvement of level V and the parotid gland (15). This could be explained by the hypothesis that our posterior tumors were generally more localized to the anterior side of the scalp in comparison with their posterior localized tumors. This thesis is supported by the fact that Vauterin et al. showed a more diffuse distribution pattern for the anterior scalp. In this case small group sizes could give a different outcome.

We did not find specific levels at risk for tumors localized at the postauricular region. On the basis of results from other studies, a selective neck dissection should be performed including level II, III and the parotid gland (15).

Tumor locations with less than 6 patients were not included in the calculation of the odds as explained in the methods section. However, the distribution of LNM from these locations was as expected.

**Table 4** summarizes which lymph nodal levels we suggest to remove at least (depending on patient specific radiological findings) during surgical resection in the case of HNcSCC with proven LNM. The percentages of the patients in this study who would have been sufficiently treated based on our advice were displayed. It should be noted that pre-operative imaging and other diagnostics of the individual patient should be included in the decision making and that the percentages presented in **Table 4** are guidelines independent of pre-operative imaging. The distribution per tumor location as depicted in **Figure 2** suggest also a more selective or conservative approach respectively, depending on the tumor location.

**Table 4:** Lymph node levels (per tumor location) suggested to be minimally included in the surgical resection, regardless of pre-surgical imaging and diagnostics of the individual patient.

Tumor location	Surgical resection should include at least:	% of patients treated sufficiently, solely due to location based advice
Ear	Parotid gland and level II	67%
Cutaneous lip	Level I ipsi- and contralateral	58%
Temporal scalp	Parotid gland	82%
Posterior scalp	(modified) radical neck dissection	67%
Ear canal	Parotid gland	57%
Postauricular	Parotid gland, level II, level III	67%

Ipsilateral unless specified.

Earlier studies only mentioned the numbers of positive levels, while we calculated odds ratios to give a more precise estimation of the probability of metastasis in different levels based on tumor location (15, 16). Furthermore, correction for regional residual disease has not been described in previous studies. Another difference is that we included patients until 2018. Improved imaging and histopathological diagnostics could therefore have played a role.

A limitation of this study is the retrospective study design and the relatively low number of patients. However, in perspective, between 2000 and 2013 770 patients were treated for cSCC in the UMCG alone (9, 22). Placing this in perspective of the low metastasis rate, one could argue that a prospective study design with a larger number of patients included would not be feasible for a single center study. Furthermore, nodal levels scored as unknown could have led to an underestimation of the involved levels. For 28 patients, an index tumor was defined, partly based on the metastasis pattern other studies described (15, 16). This could potentially have led to a stronger confirmation of this pattern. However, we primarily assessed other factors, such as time between primary tumor onset and the development of LNM, to define the index tumor.

The relatively small number of patients in our study with a clear margin after first resection of the primary tumor compared with other studies is noteworthy (7, 33). This could be explained by the nature of the tertiary care head and neck oncologic center, which has a higher number of complex tumors, including patients with positive margins after resection elsewhere. Furthermore, we only included patients with clinically suspected lymph node in comparison with most of the studies who included HNcSCC in general.

In conclusion, in this series of consecutive patients treated for LNM of HNcSCC, the known risk factors for occurrence of LNM showed no association with the extent of regional metastatic spread. However, we found a distinct metastatic pattern for specific HNcSCC primary tumor locations, such as the ear, cutaneous lip carcinoma, posterior scalp and ear canal. We provided clinical recommendations for surgical treatment of the neck and parotid gland in this emerging population. Any future predictive models for LNM of HNcSCC should include primary tumor location as an independent predicting variable.

## Data Availability Statement

The raw data supporting the conclusions of this article will be made available by the authors, without undue reservation.

## Ethics Statement

The Institutional Review Board of the University Medical Center Groningen reviewed and approved this retrospective study (IRB research register nr 202000804) and judged that there was no need for approval based on the Dutch Medical Research Law (Wet medisch-wetenschappelijk onderzoek met mensen [WMO]).

## Author Contributions

BL and BP contributed to conception and design of the study. BL and AL contributed to data acquisition. Quality control of data was performed by BV, BP and JV. Data analysis and statistics were done by BL and BD. Manuscript preparation and editing by BL. Manuscript review and additionele editing was done by AL, BD, MK, GH, GD, BV, JV, ER, and BP. Overall supervision was done by BP. All authors contributed to the article and approved the submitted version. 

## Conflict of Interest

The authors declare that the research was conducted in the absence of any commercial or financial relationships that could be construed as a potential conflict of interest.

## Publisher’s Note

All claims expressed in this article are solely those of the authors and do not necessarily represent those of their affiliated organizations, or those of the publisher, the editors and the reviewers. Any product that may be evaluated in this article, or claim that may be made by its manufacturer, is not guaranteed or endorsed by the publisher.

## Abbreviations

cSCC, cutaneous squamous cell carcinoma; HNcSCC, head and neck cutaneous squamous cell carcinoma; LNM, lymph node metastasis; University Medical Center Groningen, UMCG; AJCC 7, the American Joint Committee on Cancer 7th edition; AJCC 8, the American Joint Committee on Cancer 8th edition; BWH, the Brigham and Women’s Hospital.
